# A Unique Case of Complete Atrioventricular Block Rapidly Progressing to Diastolic and Systolic Dysfunction in Cardiac Sarcoidosis

**DOI:** 10.1155/2019/4341098

**Published:** 2019-03-06

**Authors:** Chimezie U. Mbachi, Oyintayo Ajiboye, Olisa Ezegwu, Benjamin Mba

**Affiliations:** Internal Medicine, John H. Stroger Jr. Hospital of Cook County, Chicago, IL 60612, USA

## Abstract

Sarcoidosis is a multisystem granulomatous disease that most commonly affects the lungs but can affect other organs including the heart due to granuloma infiltration. Atrioventricular block is a common manifestation of cardiac sarcoidosis which can progress to sudden cardiac death. We report a case of cardiac sarcoidosis presenting as complete heart block, progressing to diastolic and systolic dysfunction without extracardiac manifestations early in the disease. This case stresses the importance of having a high index of suspicion for cardiac sarcoidosis in patients presenting with atrioventricular block of unknown etiology.

## 1. Introduction

Cardiac sarcoidosis occurs in 20–30% of sarcoidosis patients, only 5% of them have clinical manifestations and only 40–50% of cardiac sarcoidosis patients at autopsy are correctly diagnosed during their lifetime. Diagnosis is sometimes difficult early in the disease course especially in patents without extracardiac involvement, and compared to other organs involvement, it may become fatal with a poor prognosis [1, 2].

This case highlights the importance of high index of suspicion for cardiac sarcoidosis in patients, presenting with atrioventricular block and the need for early diagnosis and treatment to possibly slow down the rate of disease progression.

## 2. Case Presentation

**Figure 1:**
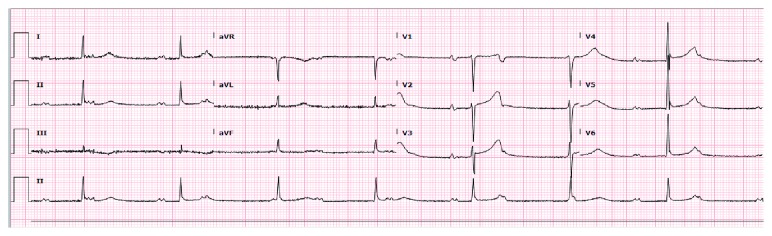
Complete AV block with junctional rhythm.

A 61-year-old African American male with medical history of diabetes mellitus presented with worsening exertional dyspnea of 2 weeks. He takes about 5 alcoholic drinks per week and quit tobacco smoking 40 years ago. On examination, he was noted to be bradycardic with heart rate (HR) 47 and hypertensive with blood pressure (BP) 180/80. Electrocardiogram (ECG) showed complete heart block and junctional escape rhythm (Figure 1). Previous ECGs were noted to show first-degree heart block and mobitz type 1 heart block, at which time he was asymptomatic. Transthoracic echocardiography (TTE) revealed an estimated ejection fraction (EF) of 55–60%, no regional wall motion abnormality (RWMA), mild increase in left ventricular wall thickness in the posterior (13 mm) and septal (13 mm) walls, and mild diastolic flattening with right volume overload. A permanent pacemaker was inserted, the ECG postprocedure showed normal electrical pacemaker rhythm. He was commenced on medications for the new onset systemic hypertension and discharged in stable condition. Of note, chest radiograph done at this admission did not reveal bilateral hilar lymphadenopathy (LAD), and LAD was also not noted on physical examination.

After a year of inadequate follow-up, the patient presented with shortness of breath (SOB) and decreased exercise tolerance (ET). No significant findings were noted on examination, and pacemaker interrogation was normal with no events recorded. Investigations were significant for mildly elevated brain natriuretic peptide- (BNP-) 248, hypoalbuminemia, and new onset normocytic anemia. TTE showed dilated right atrium, moderately reduced systolic function with an estimated EF of 30–35%, mild mitral regurgitation, moderate diffuse hypokinesis with regional variations, and grade 2 diastolic dysfunction *e*/*a* ratio of 1.7, impaired relaxation, and moderately elevated left ventricular end diastolic pressure. The patient was managed symptomatically with improvement in symptoms and discharged against medical advice (AMA). The CT angiogram chest and CT abdomen done before discharge to rule out pulmonary embolism and to evaluate an incidental finding of likely liver mass on TTE showed hepatomegaly with liver span of 17.6, innumerable nodules in the liver and spleen, with widespread lymphadenopathy (LAD) including bilateral hilar and mediastinal LAD and patchy peribronchial opacities in both lungs. These findings raised a suspicion for sarcoidosis and/or possible lymphoproliferative disorder.

**Figure 2:**
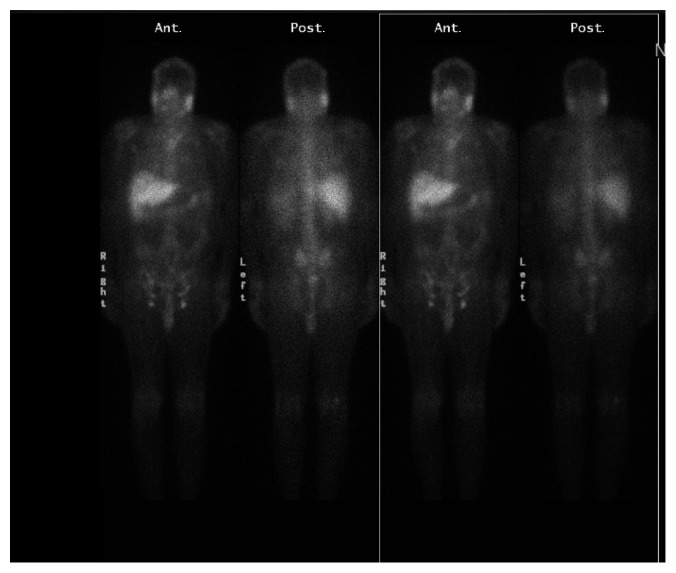
Whole body gallium scan showing increased uptake in the parotid and submandibular glands, inguinal lymph nodes, physiologic uptake in the liver, and no uptake in the chest and abdomen.

The patient did not follow-up appropriately, presented six months later with similar symptoms of SOB, and decreased ET that worsened 3 days before presentation. He becomes dyspneic on walking around in his apartment; ET tolerance was previously about 2 blocks. On examination, he was noted to have right posterior cervical and bilateral inguinal lymphadenopathy, bilateral lower extremity edema, abdominal distension, and positive hepatojugular reflux. Investigations were significant for elevated BNP-816, infiltrative pattern on liver panel-elevated alkaline phosphatase (ALP), and gamma-glutamyl transferase (GGT) with higher values compared to his last admission. TTE showed severely reduced systolic function with an estimated EF of 15–20%, severe diffuse hypokinesis with regional variations, and a severely dyssynergic right ventricle, and ECG showed short runs of native conduction with the left bundle branch block (LBBB) pattern. He was admitted and managed symptomatically for acute decompensated heart failure with systolic dysfunction. Pacemaker interrogation done revealed 88% ventricular pacing, left cardiac catheterization showed nonobstructive coronaries, and an inference of nonischemic cardiomyopathy secondary to possible cardiac sarcoidosis was made. Unfortunately, cardiac magnetic resonance (CMR) could not be done because of the pacemaker. Granulomatous disease workup done showed hypercalcemia, hypercalciuria, very low parathyroid hormone, low 25-hydroxy vitamin D, and bilateral upper lobe changes on chest X-ray. A working diagnosis of multisystemic sarcoidosis was made, and he was started on high-dose prednisone 60 mg and *Pneumocystis carinii* pneumonia prophylaxis while awaiting liver biopsy result. Whole body gallium-67 scintigraphy (Figure 2) showed increased uptake within the parotid and submandibular glands, inguinal lymph nodes, normal physiologic uptake in the liver, and no abnormal uptake in the chest and abdomen. Cardiac resynchronization therapy defibrillator (CRT-D) was done, and the patient was discharged with cardiology clinic follow-up. Liver biopsy later revealed multinucleated giant cells, noncaseating granuloma, moderate portal inflammation comprising lymphocytes, neutrophils, and plasma cells, and periportal fibrosis consistent with a clinical diagnosis of sarcoidosis. At his last clinic appointment, 6 weeks after discharge, there was significant clinical improvement as patient's symptoms had resolved, and ET improved to 5 blocks. A repeat TTE to evaluate cardiac function was planned for his next clinic appointment.

## 3. Discussion

The presentation of cardiac sarcoidosis (CS) is quite diverse and may range from asymptomatic ECG changes to sudden cardiac death. The major manifestations are atrioventricular block, ventricular arrhythmia, and heart failure [1–3]. Conduction abnormalities can be present without any evidence of cardiomyopathy; it is usually due to infiltration of the interventricular septum, leading to atrioventricular block of any degree with complete heart block as the most common. It may progress to right or left bundle branch block and sinus node arrest [1, 2, 4]. Another common manifestation of CS is heart failure, usually due to granulomatous infiltration of the myocardium causing systolic and/or diastolic dysfunction. Involvement of the myocardium may also lead to ventricular aneurysms or thinning of the ventricular free wall which may rupture in rare cases. Heart failure can also occur due to granulomatous involvement of the cardiac valves and the papillary muscles leading to mitral valve regurgitation [2, 5].

CS may be difficult to diagnose when cardiac dysfunction is the only manifestation of sarcoidosis but should be strongly suspected in patients with multisystemic sarcoidosis.

Endomyocardial biopsy is highly specific for the diagnosis of CS but it is rarely utilized because of its invasiveness and low sensitivity due to the patchy involvement of the myocardium in sarcoidosis. For diagnostic imaging, CMR and FDG-PET are preferred due to more diagnostic accuracy and higher sensitivity for detection of CS; gallium-67 scintigraphy also remains an option, although it has a lower sensitivity [3, 6–9]. CMR has an advantage over radionuclide studies as it does not expose the patient to ionizing radiation; however, it cannot be performed in patients who have implanted a cardiac pacemaker or cardioverter defibrillator [1, 2, 10]. ECG and echocardiogram may be used in the routine evaluation of patients with sarcoidosis; they are however nonspecific and nondiagnostic [1, 2]. Despite the absence of cardiac uptake on gallium-67 scintigraphy in our patient, his clinical presentation was most consistent with a clinical diagnosis of CS using the “expert consensus recommendations on criteria for diagnosing cardiac sarcoidosis” [3, 6]. The absence of cardiac uptake even with the extensive cardiac involvement in our patient was likely due to the lower sensitivity of gallium-67 scintigraphy, as reported in previous studies. Comparing FDG-PET and gallium-67 scintigraphy to the Japanese Ministry of Health and Welfare (JMHW) criteria for diagnosing cardiac sarcoidosis, the sensitivity of FDG-PET was reported to be above 80% and as high as 100% in some studies while the sensitivity of gallium-67 scintigraphy ranged between 15–37% in most studies; however, outliers were also reported [6–9].

This case highlights the progression of atrioventricular block to heart failure with both diastolic and systolic dysfunctions in CS and the need for early identification and treatment. Patients with a definite or strong probability for CS should be treated, because of its increased risk for fatal outcomes. High-dose glucocorticoid is commonly the first-line therapy; however, there is no consensus regarding dosage, treatment duration, efficacy, and use of other immunosuppressive agents, and as such, management approach varies depending on the patients presentation. Response to immunosuppressive agents is however unpredictable, and it is recommended that most patients be treated with an implantable pacemaker or cardioverter defibrillator [1, 4, 11, 12].

## 4. Conclusion

Cardiac sarcoidosis may be difficult to diagnose when cardiac dysfunction is the only manifestation of sarcoidosis. Clinicians should maintain a high index of suspicion in patients presenting with complete AV block of unknown etiology due to possibility of disease progression and fatal outcomes.

## Supplementary Materials

Supplementary 1 File 1: transthoracic echocardiography showing 4-chamber view with ejection fraction of 55–60%.

Supplementary 2 File 2: transthoracic echocardiography showing 4 chamber view with ejection fraction of 30–35%.
